# Multiple and multidimensional life transitions in the context of life-limiting health conditions: longitudinal study focussing on perspectives of young adults, families and professionals

**DOI:** 10.1186/s12904-019-0414-9

**Published:** 2019-03-25

**Authors:** Divya Jindal-Snape, Bridget Johnston, Jan Pringle, Timothy B. Kelly, Rosalind Scott, Libby Gold, Raymond Dempsey

**Affiliations:** 1 School of Education and Social Work, University of Dundee, Dundee, DD1 4HN Scotland; 2 University of Glasgow, Glasgow, UK; 3 University of Edinburgh, Edinburgh, UK; 4 University of Dundee, Dundee, UK; 5 Children’s Hospice Association Scotland, Glasgow, UK; 6 Independent Counselling Psychologist, Glasgow, UK

**Keywords:** Life transitions, Multiple transitions, Multi-dimensional transitions, Multiple and multi-dimensional transitions (MMT) theory, Young adults, Families, Professionals, Life-limiting conditions

## Abstract

**Background:**

There is a dearth of literature that investigates life transitions of young adults (YAs) with life-limiting conditions, families and professionals. The scant literature that is available has methodological limitations, including not listening to the voice of YAs, collecting data retrospectively, at one time point, from one group’s perspective and single case studies. The aim of this study was to address the gaps found in our literature review and provide a clearer understanding of the multiple and multi-dimensional life transitions experienced by YAs and significant others, over a period of time.

**Methods:**

This qualitative study used a longitudinal design and data were collected using semi-structured interviews over a 6-month period at 3 time points. Participants included 12 YAs with life-limiting conditions and their nominated significant others (10 family members and 11 professionals). Data were analysed using a thematic analysis approach.

**Results:**

Life transitions of YA and significant others are complex; they experience multiple and multi-dimensional transitions across several domains. The findings challenge the notion that all life transitions are triggered by health transitions of YAs, and has highlighted environmental factors (attitudinal and systemic) that can be changed to facilitate smoother transitions in various aspects of their lives.

**Conclusions:**

This study makes a unique and significant contribution to literature. It provides evidence and rich narratives for policy makers and service providers to change policies and practices that are in line with the needs of YAs with life-limiting conditions as they transition to adulthood. Families and professionals have specific training needs that have not yet been met fully.

## Background

### Life transitions

A change in health can trigger a non-normative life transition leading to changes in identity, status, interactions and relationships, beliefs and values; requiring substantial psychosocial and cultural adaptation [1–3]. Such life transitions require multiple lenses and any particular transition is only one part of what an individual might be experiencing [1]. In the case of a young adult (YA) with life-limiting conditions, life transitions would include their clinical trajectory, their journey from adolescence to adulthood, educational journey, changes in identity (including sexual identity), and changes in the nature and type of relationship [4]. The age and onset of health condition might affect YAs and those supporting them differently. Depending on the time and nature of diagnosis, they and their families might have to reconceptualise their entire life and aspirations. Further, the biographical uncertainty faced by these YAs can impact on their ability to engage in the psychosocial transitions required for adulthood [5]. When children with life-limiting illnesses are not expected to live to adulthood, little preparation for these transitions will have been made [4, 6].

With an increase in the number of YAs surviving paediatric life-limiting conditions [7–9], we must understand their needs and be able to effectively support them [8, 10]. It is now acknowledged that their needs will be different from when they were children [11] and that some needs will continue to be different from other YAs. The focus in the literature however seems to be mainly on clinical needs rather than holistic needs. This is perhaps not surprising as their lives are affected by their health conditions, and their transition to age-appropriate health care and adult services is still full of uncertainties and barriers [8, 12]. However, it is important to understand their holistic transitions and needs.

### YAs with life-limiting conditions: systematic literature review

A systematic literature review concerning life transitions of adolescents and YAs with life-limiting conditions was undertaken to inform the current study (Please see [13] for details). We searched MEDLINE, CINAHL, PsycINFO, CancerLit, and AMED and after several stages of searching, reviewing, including/excluding articles, a total of 18 articles were included in the review, indicating a dearth of research. This section synthesises the main findings of the literature review as relevant to this article [13].

Literature focusing on health transitions touched on YAs’ adaptation to having an illness, illness trajectory and coping strategies, including deterioration caused by illness, physical, mental and emotional, and its impact on daily activities [14, 15], such as mobility, socialisation [16], and employment aspirations [17]. However, these studies focused on the perspectives of families, with only six studies providing opportunities for adolescents to express their views.

In terms of developmental transitions, studies reported on several aspects such as employment/education/training, future planning, relationships and independence. Their future educational aspirations were constrained by the extent of their illness [18] and despite employment aspirations adolescents and YAs anticipated barriers based on their condition [17]. Similarly, some reported difficulties socialising beyond family [17], although one study found that adolescents were able to socialize with their peers after considerable organization to accommodate their health needs [18]. In one study a young person with cancer indicated that he aspired to future intimate relationships, however did not want to burden anyone with his illness [14], suggesting that normative life transitions might be constrained for those with life-limiting conditions. Some participants indicated that they had achieved illness-related independence e.g., medication and taking responsibility for healthcare [14, 18]. However, independence in other areas of their life was challenging and participants felt they were not being listened to [18].

Our review [13] also highlighted that families experienced the effect of their child’s health condition on their physical [19, 20], mental and emotional health [17, 19, 21–23]. The impact on mental health is worth highlighting as it was reported by half of the 134 parents of children receiving palliative and supportive care [19].

Although the studies provided some good insight, only six studies focused on the perspectives of the young people/YAs. The focus on proxy views is problematic as it may not be representative of the actual adolescent or YA’s views. Given their health conditions and associated needs, it is important that future research considers the best ways of collecting data directly from them. These studies reported on the impact on families’ health due to their children’s ill health but not of other transitions their child might be experiencing or other types of impacts on families. Further, impact on extended family members has not been reported, nor was the impact on professionals. Also, the impact of the transitions of families and professionals on YAs was not explored.

There were methodological limitations in these studies such as capturing the view of one stakeholder group, retrospective data collection after the death of a child, collection of data at one time point, and single case based studies. The review highlighted that very little research has taken a longitudinal approach and sought to interact with YAs over a period of time. Such an approach is crucial for facilitating the discussion of sensitive and personal issues by building rapport and an on-going relationship between the researcher and participants [24], and can provide greater depth of understanding for the researcher [25].

## Rationale, study aim and research questions

The aim of the study was to address the gaps found in our literature review and provide a clearer understanding of the multiple and multi-dimensional life transitions experienced by YAs and significant others. We have taken a holistic view of their life transitions that includes health, developmental, educational and social transitions.

Our study is underpinned by the belief, based on Multiple and Multi-dimensional Transitions (MMT) theory [1], that YAs will be experiencing multiple transitions at the same time and that this will then trigger transitions for significant others. Similarly, the transitions of significant others will trigger transitions for the YAs. Therefore, we adopted MMT theory to develop holistic understandings of the interactions between complex multiple and multi-dimensional transitions. We were mindful of this when designing this study (longitudinal, multiple perspectives) as well as during data collection (questions probing about various transitions and impacts of each other’s transitions) and analysis (looking for interactions of transitions).

To our knowledge this study is unique in three ways, a) The use of MMT in this context, b) Data collected at three time points gathering the perspectives of YAs with life-limiting conditions directly, as well as those of their families and a range of professionals, c) The YAs nominated the significant others to be interviewed.

### Research questions

(1) What multiple and multi-dimensional transitions are YAs experiencing due to their life-limiting health conditions and developmental stage? (2) What multiple and multi-dimensional transitions are significant others experiencing due to the life-limiting health conditions and developmental stage of the YAs?

## Methods

This study is underpinned by social constructionism which is grounded in the premise that meaning is constructed by people as they interact with the world [26]. The study adopted a longitudinal, mixed methods approach, using multiple sources of data collection for crystallisation of a complex and rich array of perspectives [27]. Data collection was undertaken over a 6-month period and involved serial data, gathered at 3 time points, each approximately 2 months apart.

### Ethics

Prior to applying for ethical approval, all study documents, such as participant information sheets and interview schedules, were reviewed by two parents and one YA advisor. Subsequently, ethical approval was received from the East of Scotland Research Ethics Committee (ref 14/ES/0025). The research team followed all requirements for research ethics and data protection. A thorough risk assessment was carried out, and a check-in and check-out protocol was put in place, to ensure the safety of the researcher who was sometimes working alone in the homes of participants. Pastoral support was also provided by the first author through a debriefing process.

### Recruitment

Following receipt of ethical approval, YAs meeting the inclusion criteria were contacted by two senior members of the palliative care organisation supporting them. Prospective participants were given both verbal and written information about the study. With the permission of YAs expressing an interest in taking part, their contact details were forwarded to the research team. Prior to undertaking the first interview, formal written consent was sought from young adults who were all over the age of 16. Verbal confirmation was again confirmed at all stages of data collection. During the interview process the researcher paid close attention to body language and signs of any discomfort to ensure that participants were happy to continue.

To avoid making assumptions about who their significant others were, young people were asked to nominate them at their first interview. The nominated individuals were subsequently approached, provided with participant information sheets, and asked to consent prior to their involvement. Inclusion criteria for all participants are outlined in Table 1.

**Table 1:** Participant inclusion criteria

Participants	Inclusion criteria
Young adults	• Diagnosed as having palliative care needs, and considered to be within the last 5 years of life • Aged 15–25 years • Able to provide consent • Able to communicate with researchers (through relevant means, e.g., communication aids) • Considered to be able to participate by the directors of care at the partner organisation accessed by YAs
Significant others: Family members or friends	• Family members or friends of YA above • Able to provide consent • Nominated by YA
Significant others: Professionals	• Professional staff/volunteers working with the young adults above • Able to provide consent • Nominated by YA

### Participants

#### YAs

Ten young adults took part in the first round of interviews; a further two YAs initially agreed to take part but were too unwell to participate during the first stage. However, these YAs were still interested in taking part at the time of the second stage interviews, so 12 YAs participated in the study. The age of YA participants ranged from 17 to 23 years; 9 male and 3 female. Primary diagnoses included Duchenne Muscular Dystrophy (*n* = 6), Cerebral Palsy (*n* = 2), Congenital Heart Disease (*n* = 1), other rare conditions (*n* = 3). Rare conditions are not specified to protect the identity of the participants. Geographically, participants were from rural to city locations across Scotland.

#### Significant others

Thirty people were nominated and 21 (response rate of 73%) agreed to take part (one professional was nominated by two YAs). Of the eight nominated who did not take part, four declined, and contact could not be made with a further four. The participants included 10 family members and 11 professional staff. The 10 family members included grandfather, grandmother, mother (*n* = 5), father, younger brother and older sister. Of these, the father and three mothers were fulltime carers. The two grandparents retired to take on caring responsibilities for their grandchildren.

The professionals included six social care support workers, one social worker, two nurses, one general practitioner and one consultant. They ranged in professional experience from 10 months to 20 years.

### Data collection

#### Interviews

Qualitative data were collected using semi-structured interviews with all participants. To facilitate the meaningful inclusion of YAs with communication needs, we followed the Mosaic Approach to provide a dynamic picture from multiple perspectives [28] and used needs-specific data collection techniques, such as assistive technology already used by the YAs.

Interviews took place at the participants’ convenience, mainly in their own home, but occasionally at a place of care. All YAs were interviewed on their own apart from those who required support with communication. Easy Voice Recorder and Voice Record digital recording packages were used where permission was given to audio record the interview. All interview audio recordings were transcribed by a professional transcription company. Audio recordings were sent to them, and transcriptions received, using a secure file transfer facility. One sibling and one professional declined to have their interviews recorded and hand written notes were taken. Recordings, audio or written, did not include any identifiable information.

The research team had anticipated and planned for the possibility of recruitment proving challenging. This was indeed the case with the recruitment phase of the study taking significant time. It was important that the researchers were assured that the YAs’ participation was entirely voluntary and that they did not feel obligated to take part. Table 2 details the time points of the study, the data collection methods and participants.

**Table 2:** Overview of data collection techniques, time line and participants

	Time Point 1	Time Point 2	Time Point 3
Young adults' Interviews	*n* = 10	*n* = 10 (two withdrew, but a further 2 participated)	*n* = 8 (one was unwell; one was not contactable)
Significant Others' Interviews	Nomination of family members and professionals by young adults, for data collection at time points 2 and 3	Family members, *n* = 10 Health/social care staff^a^, *n* = 10 Medical staff, *n* = 2	Family members, *n* = 4 Health/social care staff, *n* = 4

^a^includes one professional nominated by two YAs

A total of fifty-eight interviews were conducted across the three stages. After the process of introductions, consent and general familiarisation, first stage interviews with YAs ranged from 19 to 41 min in time. Interviews in stages two and three were shorter, i.e., 10–35 min. On the other hand, the interviews with family members and professionals tended to be longer, ranging from 20 to 95 min. Two YAs had minimal verbal capacity and communicated using their existing communication support and use of gestures and picture cards in line with Mosaic approach [28]. This was complemented by data from parents and professionals.

### Data analysis

Interview data were analysed using a thematic analysis approach [29]. Interview transcripts were initially read alongside listening to the audio recording for each YA and their significant others, longitudinally and as a cross-section. Although analysing the data longitudinally was invaluable to get a complete picture for every YA, results have not been presented as case studies to ensure confidentiality. A coding framework was developed and tested against each case and additional categories added as required. This process was agreed by the research team with analysis undertaken by the first author, and cross-checks by the third author.

## Results and discussion

The Multiple and Multi-dimensional Transitions theory frames the presentation of the results. Data have been presented in line with our research questions, within which we have captured the themes that emerged from the data. *Pseudonyms have been used below.*

### Multiple transitions of YAs with life-limiting conditions: educational, social and developmental (RQ1)

The YAs experienced multiple health transitions. Transitions could be from “wellness” to having a life-limiting condition, or they could be from one level of functioning or ill-health to another as conditions deteriorated or improved. Additionally, some YAs had outlived their life expectancy and were experiencing an ambiguous health status non-event (e.g., not dying). Along with health transitions, they were experiencing concurrent transitions such as educational, social and developmental. These also interacted with each other and are presented below.

#### Health and educational transitions

The data demonstrate the impact of participants’ health condition on education and future choices. Kirsty spoke about a childhood where she spent a lot of time in hospital and not keeping well.

*So when I was younger a lot of the time in hospital… I missed a lot o’ primary school…but apart fae [from] that … it doesn't really stop me, I just continue what I do…* (Kirsty, Interview 1)

She was attending a course at college and was due to finish the course a few months after the interview.

Robert talked about his post-school transitions and how his health needs and use of wheelchair were perceived as an inability to do different things.

*… I found like before I left school there wasn’t as much encouragement to find what you liked to do at college, it was almost like you're kind of pigeonholed … it's almost like eh, you're disabled…and you find that you get put in classes wi’ people with behavioural problems, or learning difficulties and … you find that and it's like you can't do what you want to do…* (Robert, Interview 1)

By the time of Interview 2 he sounded more positive about post-school choices and was looking forward to moving from the learning centre to a course at the college in his chosen area. However, Robert’s words echo the lack of options and choices for people with complex needs previously reported (see [30–32]) despite changes in legislation. Recent research suggests slight change in involvement of YAs in decision-making, but options still appeared limited with most going to further education colleges [32].

A fine-grained analysis of the data suggests that other people’s beliefs and assumptions regarding the YAs’ health condition and lack of equal opportunities/facilities might be more influential on transitional status than their actual health conditions. Alex’s example is presented in Table 3 to show this. As can be seen the diagnosis had little impact on Alex’s education and socialisation when in primary school. However, according to Alex, the size of the secondary school and moving from one class to another led to the use of wheelchair (to prevent falls). Further, assumptions about his ability based on his health condition led to the head teacher putting him in lower classes and particular subjects. This had a negative psychological impact on Alex and on his life choices.

*… so I kinda thought like I'm not smart enough to do higher stuff or something, it was kinda like, like a hint [slight laugh]… [I felt] Annoyed… ‘Cause it wasn’t my own age group, that's the problem…as soon as school finished they all went to university … and I like just went to college … I could have done it [university]… but I just never got a chance…* (Alex, Interview 1)

**Table 3:**
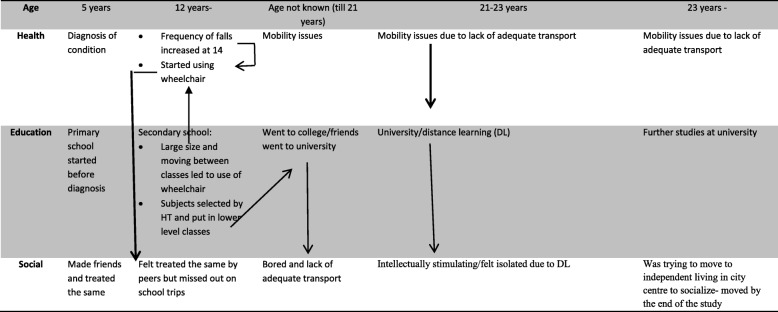
Multiple Transitions of Alex

Therefore, future educational and post-school aspirations of YAs in this study were not only limited due to the complexity and severity of their illness or barriers anticipated by the YAs as suggested by previous research [17, 18], there were systemic and attitudinal barriers that hindered their educational and employment transitions. Similarly, rather than disengaging from education or peers as reported in the case of young people with chronic health conditions [33, 34], these YAs were seen to be pro-actively creating educational and social opportunities for themselves not only at the time of the interviews but also as children.

#### Health and social transitions

Kirsty who was attending college and had her own accommodation mentioned that she was socially active.

*I've got friends at college… eh, I've got family, got carers, I've got friends here that I keep in contact with on Facebook and stuff …* (Kirsty, Interview 1)

However, similar to Palmer and Boisen’s [18] study, she highlighted the restrictions her condition placed on spontaneously going out with friends and the accommodations required for her to be able to socialise.

*… my friends that aren't disabled, they can just jump on a bus and go somewhere… I have to have somebody that takes me everywhere …* (Kirsty, Interview 1)

Similar to Abbott et al.’s study [17], Adam’s health care worker emphasised that he was not socialising with peers.

*…his social life revolves around his care staff at the moment …* (Health care professional, nominated by Adam, Interview 1)

However, through the respite care provided by a children’s palliative care organisation and other networks, some YAs, such as Susan, Harry and also Adam experienced more opportunities to socialise.

*I’m going to wheelchair football tomorrow afternoon…I’ve been going out as usual, to pictures and stuff. And out at night. Saw ‘name of a play’ at the theatre… Good access…disabled bits on different levels – bottom and top bits; I could see fine – level with the stage.* (Harry, Interview 3)

However as can be seen from Table 3, social transitions were not only affected by health transitions but also due to various decisions made by others, systemic issues to do with lack of appropriate transportation and difficult to access locations of organisations such as colleges/universities.

#### Health and developmental transitions

The developmental transitions in the context of life-limiting conditions will be presented under four sub-headings based on themes; independence, moving out, child to adult services, and death and loss.

#### Independence

The YAs in the study were reaching, or had already reached adulthood. This created some power dynamics as they asserted their independence and found some barriers (see also, [18]). Parents and care staff sometimes reported the YAs were not ready emotionally or socially to take on such responsibility, given their often protected/prolonged childhoods, and the fact that they had not previously been expected to survive to adulthood. They were, therefore, sometimes considered to be ill prepared for the adult world. Professional care staff were closely involved but were aware they were/should not be replacing family/friends. However, there was also a feeling that some parents and professionals were too controlling and over protective.

*With regard to Helen’s care team, (they need) not to micro-manage Helen… she’s the boss.* (Social Care staff, nominated by Helen, Interview 1)

*Self and sense of identity is delayed in some young people with disabilities because of the parents’ need to nurture and protect and wrap…* (Social Worker, nominated by Alex, Interview 1)

Similar to Flavelle’s study [14], there were also instances of YAs aspiring to have intimate relationships but families and/or professionals were concerned about their readiness for this.

*… he keeps saying … he wants to have sex …I think needs to come in steps, because if somebody turned up and said “let’s have sex,” he would absolutely have a heart attack… ‘cause he's not held hands with anybody…* (Health care worker, nominated by Adam, Interview 1)

*…so we thought maybe the best way was to try and get him some sort of like sex aid or sex toy or something like that* (Health care worker, nominated by Adam, Interview 2)

#### Moving out

Within the cultural context of the current study, moving out of the parental home in late adolescence/ early adulthood is normative of this transitional period; however this had extra layers of complexity for the YAs and their parents in this study. The moving out of a parent’s home signals a growing independence and a firm step towards adulthood, while it also signals a loss of role of full-time carer for the parent, triggering the family’s transition. Families found different ways to negotiate these complexities. Three examples are illustrative of the multiple and multi-dimensional transitions that one change can induce for different parts of a family system. Kirsty, at Interview 1, was already living in her own house with fulltime professional carers coming to her house. She indicated that her house provided her with control over her environment, including asking her parent or carer to leave if she wanted space. Kirsty’s parent had difficulty with adapting to the idea of her moving out, and Kirsty moved to residential facilities for a short time before securing independent housing. At the time of the interview with Kirsty, she reported her relationship with her mother was better and she saw her everyday - but on her own terms.

Living independently was also an issue in the case of Susan. Her parent negotiated with her to live at home but provided space for Susan’s boyfriend and carer.

*Susan wanted to move into a flat with her boyfriend… she's been dating since school…he’s also wheelchair-bound … I was so against it…we put the extension on the house, Susan has her own private space in here, but she's still got family support to fall back on… (name of boyfriend) comes over for sleepovers and, there's nothing more I think for her to gain from moving out, so she agreed with me...* (Susan’s parent, Interview 1)

The parent highlighted that this was only possible, and worked, as Susan was involved in choosing her carer, therefore having a sense of autonomy and ownership.

*Susan was involved in the second stage interviews, picked a girl… it works, it works, Susan has then some choice of who’s looking after her…* (Susan’s parent, Interview 1)

Finally, a recurrent theme for Alex through Interviews 1 to 3 was living independently and captured the psychological and emotional journey similar to that of Kirsty and Susan, and moving into his own accommodation by Interview 3. His social worker said that the young person moving out of the house had an impact on family in psychological terms and practical issues such as losing the mobility car and revoking of parents’ benefit appointeeship. Although from Alex and professional’s point of view this move was positive, it had a massive impact on the parent who was a fulltime carer.

#### Child to adult services

Formal health and social care systems had a profound effect on the YAs and their families in this study, especially during periods of transition. A significant systems barrier occurred in the transition from children’s services to adult services. This transition was made more complex as many of the YAs in this study were not “supposed” to live until adulthood and though adults, were still dependent on parents.

*… Children’s Services were great… anything Susan needed as a child, there wasn’t a huge fight behind it… the minute she becomes an adult it's a problem…I think in Children’s Services they understand that, that you know your child better than anyone…especially when there's communication problems* (Susan’s parent, Interview 1)

Professionals described the difficulties in adaptation for the YA (and professionals) when moving from children’s services to adult services. One (health care staff lead) highlighted this in the context of there being a fine line between the rights of James, clinical priorities and adult protection issues.

*… we have to treat him as an adult…that's quite difficult I think for him, because we’ll say no… I don’t see that as reasonable, that's going to impact on your clinical care… we maybe need tae consider if he’s refusing medication and it's causing him clinical issues, we need to take these things on-board ‘cause it's quite a complex package …* (Health Care lead, nominated by James, Interview 1)

One parent highlighted the lack of services that met her son’s needs:

*… I'm actually seeing the Social Worker … to see about getting George’s respite budget switched tae Self-directed Support… so that he can then choose where he goes … because it's difficult with his trachy stuff … not a lot o’ respite places that will accommodate that …* (George’s parent, Interview 2)

#### Death and loss

It was not clear whether families had discussed death/dying with the YAs. There were some instances of family members reporting that these discussions had not happened, such as Susan’s grandparent.

*… she went tae one o’ the funerals … she’ll talk about her friends if that's happened… gets upset and all the rest of it, but … we avoid [talking about dying]…it's down to ‘Susan’s mother’ really [to talk about it or not]* (Susan’s grandparent, Interview1)

Alex’s nominated professional highlighted the impact of parents’ transitions on Alex when they separated, and in another case the death of another YA’s father. A professional also highlighted the effect of another young person’s death as well as the sense of loss experienced when someone moves on, indicating the impact of others’ transitions on the YAs.

*… other young people … have died, and …he's been affected by those losses… also staff move on and there was a staff member died and he attended the funeral… he's… experienced a lot of death and dying*… *and… staff made redundant …so everything in his life changed… he was really sad, em and was able tae say … “what's the point in being alive when nobody really cares about you”*… (Social care staff, nominated by Philip, Interview 1)

### Multiple and multi-dimensional transitions of significant others: family (RQ2)

#### Diagnosis

Diagnosis was a stressful life transition for parents and family members. The transition to becoming a new mother was made more stressful by the diagnosis of a health condition, especially when ignored by health professionals.

*…we had a bit of a battle wi’ the, the GP tae take it seriously… I had ten months o’ going back and forward and being told I was neurotic and, a first time mother and you know he was just a lazy baby … it was quite traumatic… I was kinda getting, from my husband and my family “leave it alone, there's nothing wrong wi’ him”* (George’s parent, Interview 1)

For some the diagnosis caused a change from the identity of parent or grandparent to being a ‘carrier’. The diagnosis also brought about an anxiety or worry about off-time events. For example, Alex’s grandmother described the shock and guilt around the diagnosis as she was the genetic carrier of the condition, and concerns of him dying before her.

One grandparent, who took early retirement (another off-time transition) to support the family, described the impact of the diagnosis on the family:

*Oh traumatic … when they said that it would be unlikely she would be a teenager … and she was ten, so you're saying ‘God that's only two or three years to go,’ … traumatic, and it took us a while to get over it, as Susan’s mother said “och, I've done my grieving now,” … but… you don't, you never really do … there's still going to be an impact as well …‘cause you don't expect to have funerals for your children, never mind your grandchildren* … (Susan’s grandparent, Interview 1)

The consultant physician described the impact on Alex and family of change in survival rates.

*…they're in limbo … certainly their parents….and young lads themselves, expected them to die in their teens* (Consultant, nominated by Alex, Interview 1)

Although we did not look specifically at mental health, in line with previous research [17, 19, 21–23], we believe that there might be some signs of ‘trauma-like’ symptoms in families, and as can be seen later, the professionals.

#### Change in role and family relationships

The changing health status of the YAs created non-normative transitions for families. For example, some changes in the nature of the parental caring versus carer roles occurred. Robert spoke about the change in relationship with his parent due to his health condition and absence of professional carers.

*… it did put a strain on it for a while… me and my mum we're… quite close …and I found that the relationship was becoming, like…I was seeing my mum as a carer …and not, not my mum … you want your mum to be your mum, you don’t want her to be your carer…* (Robert, Interview 1)

Further, family relationships changed, both positively and negatively. A parent stated the impact on the sibling, Peter,

*[due to provision of extra care] I don’t have to be here... it's good because Peter and I get to spend some more time together…‘cause … missed out on so much with him and he’s very good with her… over the years…he’s been plucked out o’ bed over, overnight in pyjamas, she’s been going to hospital, he’s been put down to my mum’s or up to my brother’s and he’s just so accepting of it … I think he’s probably suffered more than anyone … ‘cause he doesn't have your normal childhood…* (Susan’s parent, Interview 1)

A sibling highlighted how the health transitions led to their parent and them becoming closer and advocates for Harry.

*… we've [Emilia and mum] always been really close … it's only ever been us two like kinda against the world … mum wasn’t particularly easy in the whole process … she was pushing people away… if they wanted to be there and part o’ her life as much as I did then they would have fought for it… [they] left us to our own devices, but we manage, we get on…* (Harry’s sister, Emilia, Interview 1)

#### Change in aspirations, social and educational transitions

For some family members, the health status of YAs made moving towards their own normative life transitions difficult/impossible or guilt provoking. For example, Susan’s brother Peter said that he was worried about the future; although willing to look after his sibling, he felt it might impede his future plans to study at university. At the time of the interview Peter was in the penultimate year of secondary school and felt plans were on hold. He found this situation stressful and although discussed with their parent, there was a great deal unknown about longevity. This resulted in Peter living with worry and uncertainty, along with concern for the parent.

Another sibling also talked about their feelings of guilt at leading a ‘normal’ life,

*At one point I was thinking, well why should I be out partying and enjoying myself, when he’s stuck in the house and he can't do nothing… why should I be going and starting a life for myself, moving away tae (name of city) …and the guilt’s probably the hardest thing because realistically you shouldn’t really have anything tae feel guilty about…* (Harry’s sister, Emilia, Interview 1)

Family members were carers and had emerging educational and training needs. Emilia described it quite succinctly.

*…my mum still bodily carries Harry … if somebody had showed her when Harry was first diagnosed how tae use a hoist and stuff like that… she probably would have used a hoist right through … I don’t understand why mothers who have got disabled children shouldn’t be put through some sort o’ care qualification tae be able tae look after their sons or daughters in the correct and proper manner, and not destroy their own bodies in the process…* (Harry’s sister, Emilia)

Harry’s parent also talked about stress:

*He has also not been able to get comfortable at night. This has a big impact on my ability to sleep, and my feelings of tiredness. It’s stressful – mental stress … he had written on his Facebook page ‘I hate my life’ … lots of emotional responses, and people contacted me with their concerns. I had to deal with all that …* (Harry’s parent, Interview 2)

Mental and physical health issues have been highlighted in previous research as well [19, 20]. However, the point made here is crucial as some carers/family members might be experiencing, avoidable, physical health problems due to lack of training.

### Multiple and multi-dimensional transitions of significant others: professionals (RQ3)

Becoming adults with paediatric health conditions resulted in educational and training needs for professionals. This is highlighted by a consultant physician who was about to retire. He stated that he was from a generation where young people with Alex’s condition only survived till their teens.

*… so not only have I had to educate myself, but … I’ve had to change my own thinking …I find that [talking about end of life issues] very, very difficult …I have never had any training or help in how [to] address these issues with young people* (Consultant, nominated by Alex, Interview 1)

Several professionals highlighted the privilege of walking with the YAs on their journeys but also indicated the emotional toll this work can sometimes take.

*… the condition he's got, you know the outcome… what to expect, but when you see it happening…it's quite heart-breaking…sometimes I’ll go home from work and I’ll just sit and, I won’t cry, but I’ll just be sitting, actually drained.* (Social Worker, nominated by Adam, Interview 1)

*…as a (name of specialisation) physician I spend a lot of time doing that [talking of death] with older people… but I find that pretty, pretty traumatic… and although I like to think that I'm a, a kind of sympathetic kind of guy…I readily admit that I feel very awkward and lacking in confidence at doing it with … YAs…* (Consultant, nominated by Alex, Interview 1)

*...it's hard, emotionally hard when you know that a young person’s … not accessing the …fundamental things of life to do with food and drink … and I guess I've been through a whole load of emotions with him…* (Social Worker, nominated by Alex)

Although not in this context, other research has highlighted the impact of such interactions for professionals, especially conflicting family and patient/client interaction, which can lead to burnout, compassion fatigue and secondary traumatic stress disorder [35, 36].

## Conclusion

### Limitations

As the sample size was small and identification might have been possible, it was considered inappropriate to present the findings as case studies. Data have therefore been presented in a generalised form rather than providing unique stories based on data from YAs and significant others in their lives. However, during the recruitment phase, care was taken to find unique cases. The quotes above related to different YAs and significant others would suggest that regardless of different contexts and circumstances, some issues and experiences were similar.

Health problems meant that a small number of YAs were not able to fully take part in the study. The study time was also shortened as a result of delays in recruitment. Despite this six of the 12 YAs took part in all three stages. Further, some participants had communication difficulties and required the use of their everyday communication aids and presence of an adult who knew the YA and their communication aid. The presence of an adult might have influenced how much and what they were willing to share.

### Implications for practice and policy

The study findings provide valuable evidence to inform both service providers and policy makers as they seek to develop services to meet the unique needs of YAs with life-limiting conditions as they make the transition to adulthood. Despite numbers appearing to be small, findings from recent research undertake in England indicate that the number of children and YAs with life-limiting conditions has doubled with as many as 32 in every 10,000 people [37] affected. In Scotland it is suggested that the numbers have risen from 75 in every 10,000 in 2009/10 to 95.7 per 10,000 in 2013/14 (Fraser et al., 2015). With growing numbers and significant needs, it is vital that services for this group are considered and well planned.

These young adults have a variety of needs related to their age and development stage. This raises the question of whether existing services, child and/or adult, are best suited to meet their particular needs. Further, we would argue that transition from children’s hospices or similar services to adult services should be based on their development *stage* rather than their biological *age*. Other organisations, in partnership with children’s hospices, might be able to provide stage related services that they were used to in children’s hospices but are not normally provided by adult services. An example would be an accessible accommodation for short planned breaks as YAs and families found this to be a really useful service offered by children’s hospices.

In this study, although YAs were at various stages, there were good examples of some YAs with life-limiting conditions living independently. However, it was clear that for this to happen, they required services to focus on them as a whole, with provision of medical, psychosocial, and educational support for them and their family. As transition is an on-going process, it is important that transition planning and preparation is on-going too.

A number of family members in this study reported that they did not feel they had appropriate training to effectively manage YAs’ care needs. As the immediate and stable support network of the YA, it is particularly important that families are provided training; and that this training is on-going congruent to YAs’ stage and age needs. Further, in line with the MMT theory, it is crucial that family members recognise their own transition needs and prepare early in anticipation of the impact of their child/sibling’s transitions. Similarly, the professionals should be mindful of the transition support needs of families to effectively support them. This also highlights the importance of *all* health and social care professionals receiving relevant training so that they can signpost and advise YAs and families on how to access resources for effectively supporting transition into optimum pathways.

Again, in line with the MMT theory, there is evidence that professionals can also be affected by the YAs’ transitions, with some reporting ‘stress-like’ symptoms. Those professionals working in organisations seemed to have supervision and debriefing available to them; however it was not clear what support and supervision systems are available to professionals employed by families. Therefore, it is important that there is further consideration of support and supervision systems for professionals at policy and practice levels.

### Implications for future research

This study provided unique insights due to interviews being conducted at three time points. Although this was resource intensive, we recommend that future research follows a similar research design, with a larger sample, and over a longer period. This should provide the option of constructing case studies without compromising identification of participants, and being able to capture further changes over time.

As none of the YAs nominated educational and careers staff, it was not possible to explore the opportunities available for educational and work transitions. In future, it would be useful to include the perspectives of a wider range of service providers, such as school teachers and guidance staff, college/university staff, voluntary organisations, and careers advisors to better understand the opportunities and removal of any barriers to enable YAs to realise their aspirations.

The data suggested potential ‘stress-like’ symptoms for families and professionals. This should be investigated further, firstly to understand whether this is the case and secondly if that is the case, what are their related support needs. Further, a training needs assessment should be undertaken.

### Original contribution and significance

This study was unique in that it captured the voices of YAs with life-limiting conditions, including those with very little functional verbal communication. Further, the data were captured over time, and went beyond the focus of previous studies on health transitions by situating it within holistic life transitions. It also looked at the impact of one transition on other transitions. It challenges the notion that all life transitions are triggered by health transitions, and has highlighted attitudinal and systemic factors that can be changed to facilitate smoother transitions. Also, as anticipated, we found that transitions of others (e.g., family members, peers and professionals) have the potential to trigger other transitions for them [1].

Similarly, we found that significant others were experiencing multiple and multi-dimensional transitions, some of which were linked with the YAs’ transitions [1]. For some, there was evidence of holding back from certain transitions and/or feeling guilty about moving on when the YA was unable to do so or required their support. Others had speeded up their transitions such as in the case of people who had retired early or stopped working to look after their child/grandchild.
